# Exploring the surveillance technology discourse: a bibliometric analysis and topic modeling approach

**DOI:** 10.3389/frai.2024.1406361

**Published:** 2024-06-06

**Authors:** Kalle Karlsson, Fisnik Dalipi

**Affiliations:** Department of Informatics, Faculty of Technology, Linnaeus University, Växjö, Sweden

**Keywords:** topic modeling, machine learning, surveillance technology, social media, security, privacy

## Abstract

The prevention of crime is a multifaceted challenge with legal, political, and cultural implications. Surveillance technologies play a crucial role in assisting law enforcement and other relevant parties in this mission. Drones, cameras, and wiretaps are examples of such devices. As their use increases, it becomes essential to address related challenges involving various stakeholders and consider cultural, political, and legal aspects. The objective of this study was to analyze the impact of surveillance technologies and identify commonalities and differences in perspectives among social media users and researchers. Data extraction was performed from two platforms: Scopus (for academic research papers) and platform X (formerly known as Twitter). The dataset included 88,989 tweets and 4,874 research papers. Topic modeling, an unsupervised machine learning approach, was applied to analyze the content. The research results revealed that privacy received little attention across the datasets, indicating its relatively low prominence. The military applications and their usage have been documented in academic research articles as well as tweets. Based on the empirical evidence, it seems that contemporary surveillance technology may be accurately described as possessing a bi-directional nature, including both sousveillance and surveillance, which aligns with Deleuzian ideas on the Panopticon. The study’s findings also indicate that there was a greater level of interest in actual applications of surveillance technologies as opposed to more abstract concepts like ethics and privacy.

## 1 Introduction

Technology has become an important part of modern life; it is pervasive and is exhibiting continuous advancements and a simultaneous decrease in cost. This phenomenon has led to expanded possibilities for governmental bodies to incorporate technology in many domains. Furthermore, there is an increasing prevalence of people using information and communication technology (ICT), including telecommunications technology and social media, to a greater extent. In the present era of technological advancements, individuals are afforded many opportunities to continuously create data. Hence, it can be observed that a substantial volume of data is created continuously on a global scale. One of the significant contributors to the overall data volume is the substantial quantity of video data created. Various sectors, such as education, healthcare, tourism, culture, geographical exploration, agriculture, safety and security, and entertainment, create a tremendous volume of video data on a daily basis (Afzal et al., 2023). A significant portion of the gathered data consists of surveillance data obtained from security cameras that is utilized on a regular basis and recorded every day (Tan et al., 2022; Vosta and Yow, 2022; Huszar et al., 2023). Undoubtedly, such data is also used for the purpose of implementing preventive and incapacitating strategies to address and alleviate criminal activities.

Various surveillance technologies are developed for the storage, retrieval, processing, and analysis of these data. Surveillance technology ranges from wiretaps and cameras to cutting-edge drones and satellites (Cayford and Pieters, 2020). Subudhi et al. (2019) argue that the convergence of surveillance technologies and big data analytics has sparked considerable interest in the field of surveillance discourse. One cannot overlook the significance of integrity and privacy concerns surrounding prominent corporations like Google and Facebook. Moreover, the surveillance environment is increasingly influenced by technologies such as the Internet of Things (IoT) and Artificial Intelligence (AI). One crucial concern pertaining to these technologies, however, is their impact on crime prevention efforts (Thomas et al., 2022). These inquiries may relate to the efficacy of such measures, their user-friendliness, and the potential adverse impact on individuals, such as the violation of their privacy. According to the renowned sociologist Anthony Giddens, surveillance may be classified into two distinct categories: the collection and storage of encoded data, and the exercise of direct oversight by governing bodies (McCahill, 2002).

The use of digital technologies along with surveillance techniques has also shown considerable efficacy in previous instances of disease epidemics, including cholera and Ebola (Gupta and Katarya, 2020). Nevertheless, there have been concerns regarding the potential infringement on personal privacy due to the gathering of personal data through contact tracing apps, such as real-time location tracking, surveillance of online behaviors, such as monitoring Google search queries, and the use of biometric authentication methods, such as thermal cameras employed for body temperature checks in airports and other locations. This issue becomes a topic of controversy and garners public interest in relation to the protection of individual privacy rights. Concerns have been raised by privacy experts over the potential future normalization of surveillance techniques and monitoring systems (Calvo et al., 2020). There is a significant level of ambiguity around the potential future use of the aggregated data after the COVID-19 epidemic emerged. Considerable research has been performed to investigate the issues surrounding privacy concerns and information disclosure in commercial contexts involving online providers. However, there is a noticeable lack of research on public perspectives and attitudes toward surveillance technologies (Reddick et al., 2015), and the subsequent impact on individuals’ privacy-related behaviors (Dinev et al., 2008; Nam, 2019). Given the increased significance of the matter, especially during COVID-19 pandemic, it is crucial to get a deeper understanding of individuals’ perspectives on data collection and surveillance technology.

The objective of this study is to examine the topic of surveillance technology and delineate the commonalities and differences in perspectives between social media users and scholars. Thus, the present study aims to address the following research inquiries:

RQ1: What are the research trends pertaining to surveillance technology, and which patterns can be observed across various topics?

RQ2: Which similarities and discrepancies can be found in research papers and the public discourse on social media platform X (previously known as Twitter)?

The paper is organized as follows: In the next section, we provide the theoretical framework and literature review, followed by methodology. In the fourth section, we present the results of our research, whereas section five includes discussion with regards to theoretical and practical implications. Section six concludes this work.

## 2 Theoretical framework and related work

### 2.1 The theory of Panopticism

The notion of the Panopticon serves as a recurring and significant metaphor for understanding the paradigms of surveillance and the theoretical frameworks that underlie them, as supported by noteworthy works (McCahill, 2002; Galič et al., 2017; Ceccato, 2019). The notion of the Panopticon, which originated in the 18th century, became widely known in the 1970s, mainly due to Michel Foucault’s book “Discipline and Punishment: The Birth of the Prison” (Caluya, 2010). Foucault, a prominent historian, focused his academic investigations on the complex interplay between power and knowledge, asserting their inseparable interdependence and so introducing the fundamental concept of “power/knowledge.” The primary focus of his theoretical corpus of work mostly revolved on the many dimensions of power as it is expressed within the context of social control, often shown via governmental agencies and institutions.

Although Foucault’s corpus of work is not directly relevant to the field of information systems (IS), it has made noteworthy contributions. This statement pertains to the author’s viewpoint of the coexistence of technology in close proximity to human beings, irrespective of their conscious recognition of such coexistence (Willcocks, 2004). This concerns the socio-technological aspects of information systems as well as the prevailing existence of technology in contemporary society. In addition, Foucault contends that it is unsuitable to see technology as having deterministic attributes. Closed-circuit television (CCTV) surveillance technologies are often associated with the Panopticon concept. According to McCahill (2002), the notions of the Panopticon are intrinsically linked to the perspectives of individuals inside society as well as the institutional frameworks in which they operate. Foucault’s use of the Panopticon metaphor was only a component of a broader endeavor to comprehend the intricacies of power (Caluya, 2010). In addition, the author argues that in modern society, it is necessary to complement Foucault’s position with other viewpoints, especially due to the evolving nature of technology. Consequently, there has been a transition toward Deleuzian examinations that prioritize a nuanced and multifaceted perspective on surveillance, whereby networks of individuals interact, as opposed to a more fixed understanding of the Panopticon.

According to Galič et al. (2017), recent scholarly investigations have introduced novel insights into the interplay of power dynamics in response to changes occurring within the socio-technological environment. Regarding theory, Foucault explicitly expressed that he was engaged in “analytical work” rather than theory. He also identified parallels with the German philosopher Nietzsche’s observation that while thinkers constantly throw arrows into the air, the crucial aspect is for others to intercept and redirect them in a different direction (Willcocks, 2004). However, his contributions have traditionally been used by researchers in the field of IS for theoretical direction and remain relevant to contemporary technologies like CCTV. This demonstrates the influence of Panopticon beyond its initial physical context, namely in public areas. According to Foucault’s prediction, panopticism has certainly permeated non-institutional places and populations (McCahill, 2002). Ultimately, it is possible that the Panopticon metaphor has been seen as biased, and it is more appropriate to engage with Foucault’s work rather than just imitate it. According to Willcocks (2004), Foucault may be used to analyse the relationship between control, certainty, and knowledge in the context of human use of ICT. This analysis allows for a broader understanding of ICT within society, without being limited to its hardware and software aspects.

The Panopticon metaphor is used in this study to analyse and explore the empirical results. Based on the empirical evidence, this study aims to assess the extent to which the Panopticon metaphor remains applicable in the contemporary technological environment, characterized by significant advancements and widespread use of technology.

### 2.2 Related work

An analysis of relevant scientific literature was conducted to assess the current state of knowledge on this topic. These academic publications jointly examined the complex field of surveillance technologies from a socio-technological standpoint. The papers chosen for reference in Table 1 are mostly drawn from relevant journals and include the latest achievements and insights.

**Table 1:** Articles reviewed.

Article	Journal
Brayne (2017)	American Sociological Review
Cayford et al. (2020)	Journal of Information, Communication and Ethics in Society
Fussey (2004)	Surveillance and Society
Hassan et al. (2021)	International Journal of Information Management
Kumar and Venkateswara Reddy (2022)	Concurrency and Computation: Practice and Experience
Shih et al. (2019)	Symmetry
Su et al. (2022)	Journal of Information Technology & Politics
Suarez-Paez et al. (2019)	Information
Sun (2020)	Journal of Visual Communication and Image Representation
Sung and Park (2021)	Multimedia Tools and Applications
Van der Vlist (2017)	Surveillance & Society
Custers (2012)	Computer Law and Security Review
Galič et al. (2017)	Philosophy & Technology
Fox et al. (2021)	Computers in Human Behavior
Blythe et al. (2004)	Personal and Ubiquitous Computing
Nunn (2001)	Technology in Society
Koper et al. (2015)	Evaluation Review
Ceccato (2019)	Criminal Justice Review

The process of systematically analyzing articles enabled the discovery of recurring themes, which were then used to classify and organize the material under evaluation. The themes that were discovered had a double function: firstly, they assisted in the initial selection of research topics as described in the methodology section, and secondly, they served as the foundation for the analysis and contextualization of the results. Furthermore, the topics that have been identified provide a complete overview of the extensive research that has been undertaken on surveillance technologies within the field.

#### 2.2.1 Themes

**Figure 1:**
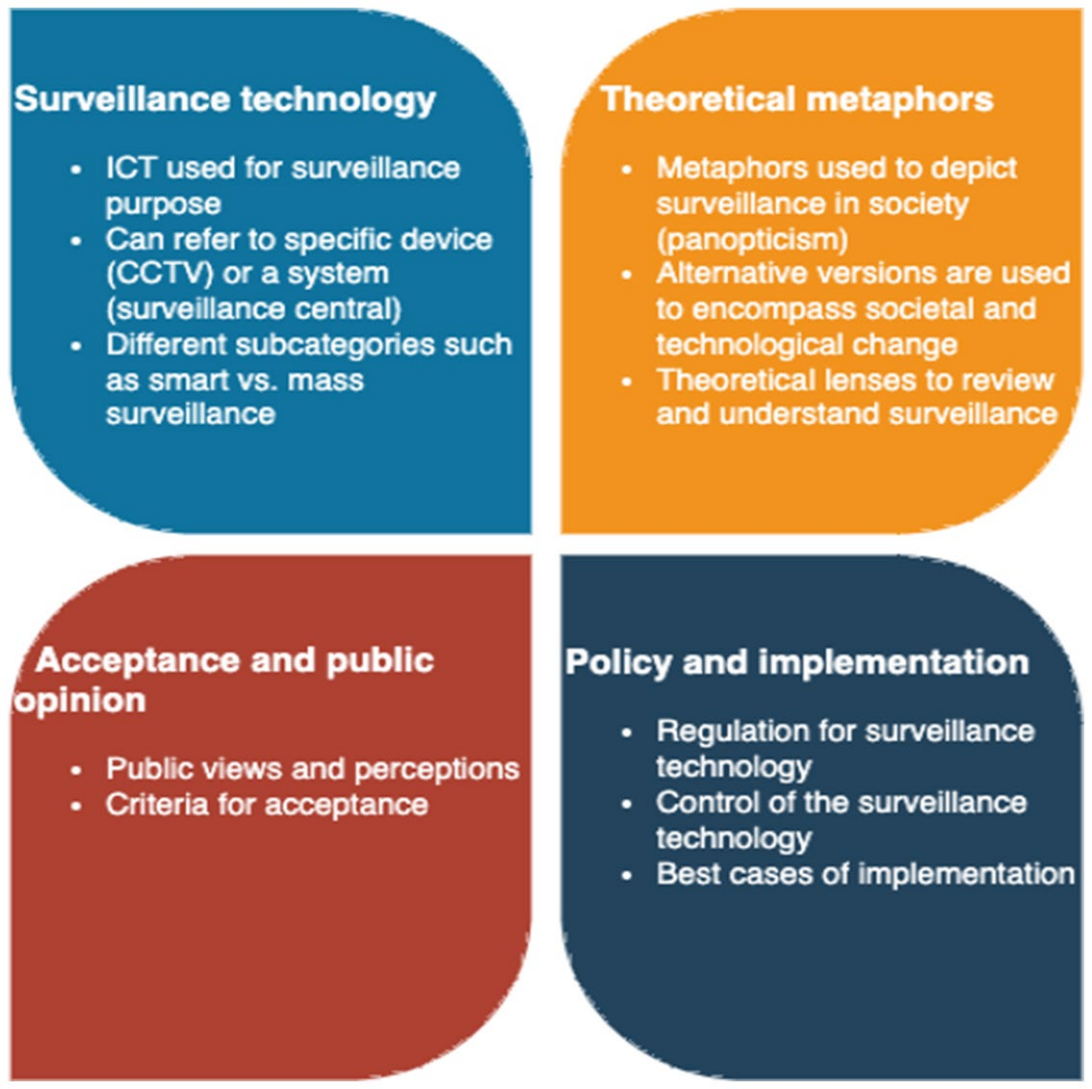
Identified themes.

Based on the reviewed articles four themes were discerned in the state-of-the-art literature. These are presented in Figure 1 and elaborated in subsequent subsections.

##### 2.2.1.1 Surveillance technology

Etymologically, the term ‘surveillance’ refers to a historical perspective from a higher position, originating from the combination of ‘sur’ (from above) and ‘veillance’ (to observe). The breadth of technological improvements, particularly in the field of Information and Communication Technology (ICT), has expanded beyond conventional uses such as public cameras (Galič et al., 2017). The prevalence of traditional surveillance equipment has been growing, but the incorporation of other technologies like drones and face recognition indicates a broadening scope (Fox et al., 2021). Contemporary applications include the use of real-time data for the purposes of law enforcement and disease monitoring. This aligns with the findings made by Custers (2012) on the interest of law enforcement in face recognition, drones, and Virtual Reality (VR) technologies, despite the absence of significant validation for their effectiveness. Biometrics, monitoring, imaging, decision assistance, record-keeping, and armament are among the several instruments used in law enforcement (Nunn, 2001). The use of machine learning in smart video surveillance has the potential to improve the reaction of law enforcement to occurrences (Sung and Park, 2021), hence solving the problems and resource limitations associated with human criminal detection. According to Slobogin (2008), state monitoring may be divided into two categories: physical surveillance and transactional surveillance. CCTV is an example of physical surveillance, while data analysis of video is an example of transactional surveillance. The widespread phenomenon of monitoring, referred to as ‘mass surveillance’ by Brayne (2017), gives rise to apprehensions over the protection of individual rights (31). It is worth noting that current surveillance trends place significant emphasis on the significance of ICT, often functioning inconspicuously (Van der Vlist, 2017). Furthermore, the notion of ‘sousveillance’ highlights the possibility for people to use surveillance instruments, hence requiring discretion to avoid their misuse and the dissemination of inaccurate information (Shih et al., 2019).

##### 2.2.1.2 Theoretical metaphors (meta-knowledge)

In the landscape of contemporary society, human surveillance transcends physical and digital boundaries, demanding adaptable theoretical frameworks for comprehensive research. The Panopticon metaphor, historically foundational to surveillance theory, epitomizes continuous observation without detainees’ awareness, originating from Jeremy Bentham’s utilitarian ideals (Galič et al., 2017; Ceccato, 2019).

The evolution of surveillance theory spans three historical periods:

1. Physical constraints of surveillance: represented by Foucault and Bentham’s Panopticon, emphasizing surveillance’s limitation by physical barriers.
2. Abstract perspectives emphasizing networks: shifts focus on networks over institutions in surveillance discourse.
3. Integration of physical and digital surveillance: encompasses dataveillance and access control, reflecting contemporary surveillance in both physical and digital realms.

Neo-Marxist examinations of surveillance mostly focus on the two functions of closed-circuit television (CCTV), which include the policing of marginalized communities and the representation of state power. This aligns with Foucault’s emphasis on surveillance as a symbol of authority (Fussey, 2004). Modern surveillance terms, such ‘eyes on the street’ and ‘sousveillance,’ refer to the complex situation where personal technology is used for both intentional and hidden monitoring, reflecting the fundamental principles of the Panopticon in contemporary society (Ceccato, 2019). The fast advancement of ICT poses a challenge to the conventional one-way surveillance structure, requiring a reevaluation of surveillance dynamics. The reciprocal nature of surveillance is becoming more evident in our technologically evolved society and is expected to increase with additional improvements in information and communication technology (Thomas et al., 2022). Foucault’s concept of Panopticism transcends the limits of prison architecture, serving as a representation of the ubiquitous power of monitoring that shapes social norms and dynamics (Galič et al., 2017). This serves to illustrate the enormous impact of surveillance across many domains within society.

##### 2.2.1.3 Acceptance and public opinion

An essential aspect in this research is recognizing the crucial influence of public views and their willingness to adopt monitoring technology. This characteristic is of particular importance in democratic systems, where policy is shaped by public opinion, as opposed to authoritarian regimes where citizen perspectives have relatively less influence. A study carried out by Su et al. (2022) brought attention to significant approval among Chinese individuals for several manifestations of state monitoring. The importance of careful interpretation of these data is underscored by the authoritative character of the Chinese state and the inherent limits in survey dependability. However, this research highlights the significant impact of public approval on the acceptance and utilization of surveillance technology, while acknowledging that China’s unique circumstances may be relatively uncommon in this aspect. The research conducted by Cayford et al. (2020) aimed to investigate public opinions on the effectiveness of surveillance technology, with a specific emphasis on privacy issues. Surprisingly, their research found that people do not regard a compromise between efficacy and privacy. In contrast, it seems that people place importance on both the effectiveness and cost-efficiency of monitoring techniques, while also ensuring the protection of privacy against violation. Nevertheless, the research divides people into two overarching groups: (i) those who demonstrate trust in surveillance technology to operate without causing any disruption while accomplishing its goals, and (ii) individuals who challenge its efficacy and see it as infringing upon privacy limits.

##### 2.2.1.4 Policy and implementation

The effect of policy and its execution is a crucial aspect of surveillance technology, particularly when seen through the lens of information systems. Soft systems technique, used by IS practitioners and academics, is a method that is relevant in this context (Checkland, 2000). According to Fussey (2004), the creation of CCTV policies is significantly influenced by the extent to which practitioners at the local level comprehend and adhere to them. The extent to which these policies vary is contingent upon regional and cultural settings, which are essential factors to consider throughout the process of their implementation. Fussey (2004) further asserts that the attainment of compliance from local practitioners necessitates the use of more effective methods beyond simple persuasion. This underscores the significance of local processes, institutions, and agency in shaping the creation of CCTV policies. The predominant focus of surveillance technology policy seems to be on its efficacy rather than ethical considerations related to privacy and integrity (Custers, 2012; Hassan et al., 2021). This statement does not imply that these factors are not considered, but rather emphasizes a stronger focus on efficacy. According to Cayford et al. (2020), it is possible that efficacy and ethics are closely connected. The proficient use of non-arbitrary monitoring technology is associated with less infringement on privacy, and vice versa. Hassan et al. (2021) argue that conducting criminal investigations at a minimal expense is a crucial element, and that formulating policies based on data might serve as a means to succeed. Suarez-Paez et al. (2019) emphasizes that it is crucial to minimize computational expenses in order to ensure the financial viability of monitoring system installation.

## 3 Methodology

**Figure 2:**
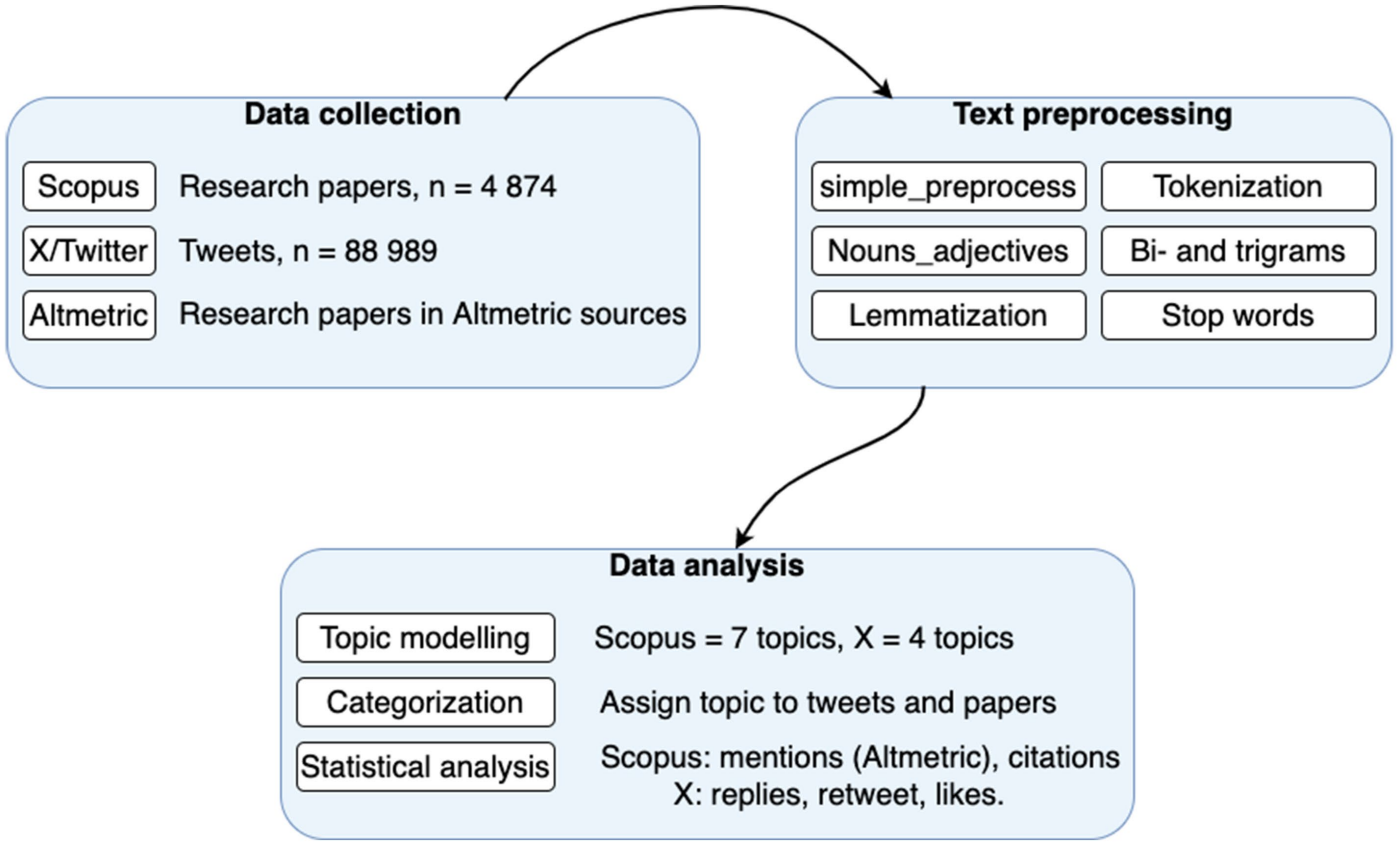
Methodological workflow.

This section describes how data collection and data analysis was carried out. The methodological workflow used in this study is presented in Figure 2 and explained in the next subsections.

### 3.1 Data collection

#### 3.1.1 Research papers

The Scopus database was used for the purpose of accessing and retrieving research articles due to its status as a comprehensive citational database that encompasses a wide range of academic journals. This method was used to export 4,874 research articles in batches of 2000. The search query used to obtain studies was as follows: TITLE-ABS-KEY ((surveil* AND (tech* OR ict OR telecommunication* OR "information technolog*" OR "internet of things*" OR "Information and communication technolog*" OR "computer technology" OR computing OR "big data" OR "data analysis" OR "machine learning" OR ai OR datalogy OR "artificial intelligence" OR cybernetics OR data)) AND (crime* OR criminal* OR "law enforcement*" OR police* OR policing*))

#### 3.1.2 X (Twitter) platform

To obtain full access to X/Twitter data, we underwent the application and verification process for the utilization of X/Twitter’s academic research API. However, it’s essential to acknowledge inherent trustworthiness challenges on X/Twitter, predominantly due to its nature as a platform primarily driven by user-generated content (Reuter and Kaufhold, 2018). Notably, within the scope of this paper, the focus is less on the veracity of individual information and more on the analysis of collective public viewpoints. The extraction of tweets was conducted through API-calls utilizing Python. The search query to fetch the tweets is shown below. search_query = '(((surveil OR surveillance) AND (tech OR technology OR ict OR telecommunication OR "information technology" OR "internet of things" OR "Information and communication technology" OR "computer technology" OR computing OR "big data" OR "data analysis" OR "machine learning" OR ai OR datalogy OR "artificial intelligence" OR cybernetics OR data)) AND (crime OR criminal OR "law enforcement" OR police OR policing)) lang:en -is:retweet -is:reply'

This approach yielded a total of 88,989 tweets.

#### 3.1.3 Documents and Altmetric mentions

In order to assess the impact of research papers beyond academic circles, Altmetric.com served as a primary resource. Access to the API was obtained for research purposes, enabling unrestricted queries and a comprehensive analysis of research paper citations across various altmetric sources, encompassing social media, policy documents, and news outlets. Querying the database involved utilizing the DOIs of research papers to retrieve response counts of mentions.

This study utilized specific fields, including *‘title’, ‘cited_by_accounts_count’, ‘cited_by_posts_count’, ‘cited_by_msm_count’, ‘cited_by_policies_count’,* and *‘cited_by_tweeters’*. The findings were presented through quantitative measures illustrating the dissemination of altmetric impact. Furthermore, a qualitative examination of the most frequently mentioned papers provided insights into the nature and context of altmetric mentions.

### 3.2 Data analysis

This section outlines how data analysis was done which entailed preprocessing tweets and research papers and then analyzing using topic modeling.

#### 3.2.1 Text preprocessing

The Gensim function *`simple_preprocess`* was used as a preprocessing tool, transforming raw text into a consistent sequence of lowercase tokens by excluding non-alphanumeric characters and delimiters. This utility employs tokenization to segment input text into individual words or tokens, standardizing their case for uniformity. With configurable parameters, it enables the removal of non-alphanumeric characters and grants control over token length, streamlining the conversion of textual data into a structured format ideal for subsequent natural language processing endeavors, including text analysis, topic modeling, and document classification.

Moreover, the text underwent specific preprocessing steps involving word filtration based on parts of speech, retaining only nouns and adjectives. Lemmatization was performed using the *`data_lemmatized`* function, further refined the words to their base forms. Token acceptance criteria were confined to alphanumeric characters and the symbols ‘_’, ‘#’ within words, with a stipulated minimum word length for each dataset.

Additionally, the process incorporated the creation of bi- and trigrams, identifying meaningful phrases consisting of multiple words (e.g., “real time”) to capture nuanced contextual information embedded within the text. Within the given setup, the minimal frequency of occurrence of a word is 15 (Scopus) and 100 (X/Twitter). Stop words are derived from the English language and have been used as a delimiter during the retrieval of research articles and tweets. Topic modeling code snippets written in Python are provided in the Supplementary material.

#### 3.2.2 Topic modeling and LDA

Topic modeling, a machine learning technique, serves to unveil underlying themes within textual data by algorithmically analyzing word distributions (Cain, 2016). It involves clustering words to identify patterns, drawing from a computational paradigm while necessitating human interpretation, thus intertwining positivist and interpretivist influences (Mimno et al., 2011; Sievert and Shirley, 2014).

Latent Dirichlet Allocation (LDA), a widely used probabilistic modeling technique in natural language processing, unveils latent structures within text corpora (Blei et al., 2003). The primary objective of the LDA model is to find the best way for creating a new document from each raw document while also increasing the probability, which is shown in the equation below.


$$\begin{array}{l} p\left(\omega ,\;z,\;\theta ,\;\eta \;|\;\alpha ,\beta \right)=\overset{M}{\underset{d=1}{\prod }}p\left(\theta_{d}\;|\;\alpha \right)\overset{K}{\underset{k=1}{\prod }}p\left(\eta_{k}\;|\;\beta \right)\overset{N}{\underset{t=1}{\prod }}p\left(z_{dt}\;|\;\theta_{d}\right)\\ \;p\left(\omega_{dt}\;|\;\eta z_{dt}\right) \end{array}$$

where *α* and *β* represent Dirichlet distribution parameters, *θ* and *η* are the multinomial distributions, *z* indicates the defined topics in all documents, *ω* denotes the entire set of words present in all documents, and *M*, *K*, and *N* indicate the number of documents, number of topics, and the number of words, respectively.

Operating under the premise that documents comprise a mixture of latent topics, LDA estimates these topics iteratively based on word co-occurrence patterns (Blei, 2012). Each word in a document is probabilistically assigned to a latent topic, highlighting the thematic structure across the collection without predefined labeling (Blei and Lafferty, 2006). LDA’s versatility spans exploratory analysis, topic modeling, and content summarization (Blei, 2012).

Within this study, Python was employed to script topic modeling, necessitating meticulous parameter tuning, particularly the number of topics and training set division. Parameters controlling algorithm analysis and iteration dynamics, such as *learning_method, learning_decay*, and *batch_size*, were adjusted. Custom stop words were tailored for distinct datasets like Scopus and X/Twitter due to language and terminology disparities. For instance, in the Scopus dataset, stop words such as *“surveillance,” “technology,”* and *“surveillance technology”* were excluded due to their frequent occurrence and limited discriminatory value in discerning distinct topics. In contrast, the X/Twitter dataset required a different set of stop words, including *“surveillance,” “technology,” “surveillance technology,” “police,” “http,” “new,” “use,” “https,” “tech,”* and *“say..”* These terms were identified as common but lacked the granularity necessary for meaningful thematic differentiation in the X/Twitter dataset, thus being omitted from the analysis to enhance the modeling process. Iterative model refinement employed a grid-search function complemented by human judgment, and *LDAvis* tool facilitated visualization of topic coherence. Consequently, the Scopus dataset yielded seven topics, whereas X/Twitter yielded four. This demonstrates the influence of entity format, language, and terminology variations on the outcome. One example is the diverse use of stop words in the datasets. The selection of topic labels was determined by the authors’ knowledge in the area of IS, supported by insights derived from the carefully examined publications. The *LDAvis* interface was used to thoroughly examine each topic, identifying the 30 most important words for each topic. These words were then used to create the topic labels.

#### 3.2.3 Computation of measures in findings

Weight was computed by dividing the share of the chosen topic within each data point. One such instance is shown in Table 2 below. For row 1 the weight of topic 0 is 0.72 and for row 2 the weight of topic 1 is 0.76. In the data retrieved the weight of all entries for each topic were calculated into a joint score for the whole topic.

**Table 2:** Frequency and weights (a representative example).

Topic0	Topic1	Topic2	Topic3	Topic4	Topic5	Topic6	Dominant_topic
0.72	0.27	0	0	0	0	0	0
0.01	0.76	0.,01	0.01	0.2	0.01	0.01	1

### 3.3 Ethical considerations

The commitment to the established norms defined by the Swedish Research Council (2017) has been of utmost importance throughout the whole of the research endeavor. These principles provide detailed recommendations that outline procedures, with a focus on promoting best practices for reference methodologies. Moreover, in accordance with these ethical principles, the codes used in this research are widely disseminated, according to the principles of open-source customs. The objective of this approach is twofold: firstly, to facilitate the widespread distribution of information, and secondly, to improve the level of transparency in the approaches used in this study.

Furthermore, the implementation of the Twitter Academic Research API precisely aligns with its designated objective, with a special focus on supporting ethical and non-commercial academic research initiatives. In compliance with ethical research norms, the user data obtained via this API is pseudonymized, thus safeguarding confidentiality and privacy.

## 4 Results

### 4.1 Research papers (Scopus dataset)

**Figure 3:**
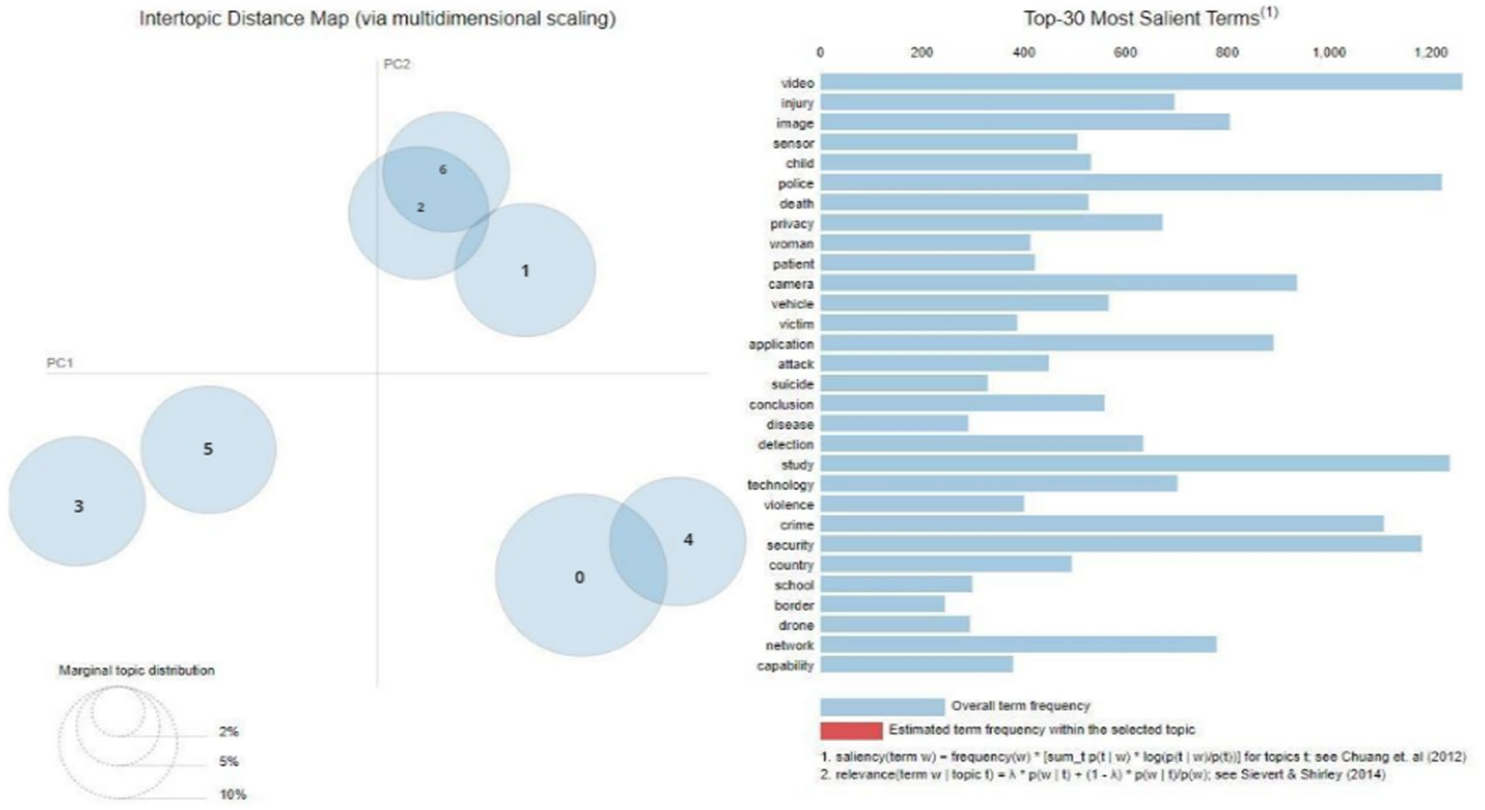
Topics from Scopus.

By using LDA topic modelling, a total of seven unique topics were identified, as seen in Figure 3. Additionally, this figure presents a graphical depiction of the 30 most prominent terms linked to each respective topic.

The visual depiction in Figure 3 showcases a very even distribution of topics exhibiting comparable sizes and spread across the chart, but there are some instances of overlap among a few topics. The topics identified in the corpus include privacy, sensor, detection, camera, drone, and video. Table 3 provides a detailed overview of the top 15 keywords that define each determined topic.

**Table 3:** Topics (Scopus dataset).

Number	Label	Keywords
Topic 0	E-health & disease management	Study, patient, country, disease, program, result, population, method, health, region, datum, conclusion, number, analysis, treatment
Topic 1	Policing & monitoring	Injury, datum, child, death, woman, victim, suicide, conclusion, result, study, violence, school, method, state, police
Topic 2	Privacy & Security (market and private sector)	Information, security, datum, technology, internet, privacy, practice, communication, process, service, digital, device, analysis, individual, market
Topic 3	Privacy: Regulation & Legislation (Societal level)	Privacy, crime, state, policy, government, individual, legal, court, right, protection, criminal, issue, measure, offender, regulation
Topic 4	Policing and law enforcement	Police, crime, study, space, border, technology, power, social, author, policing, political, practice, officer, community, control
Topic 5	Applications for surveillance technology	Video, image, camera, technique, method, paper, detection, model, problem, application, feature, real_time, crime, object, analysis
Topic 6	Defense & military implementation	Sensor, application, vehicle, attack, network, security, operation, capability, drone, paper, military, design, threat, radar, development

Naturally, each topic relates to surveillance technology to mitigate and combat crime in one way or another but when reviewing each topic and its keywords the findings show that there are distinct perspectives to be discovered in research papers on the subject.

**Figure 4:**
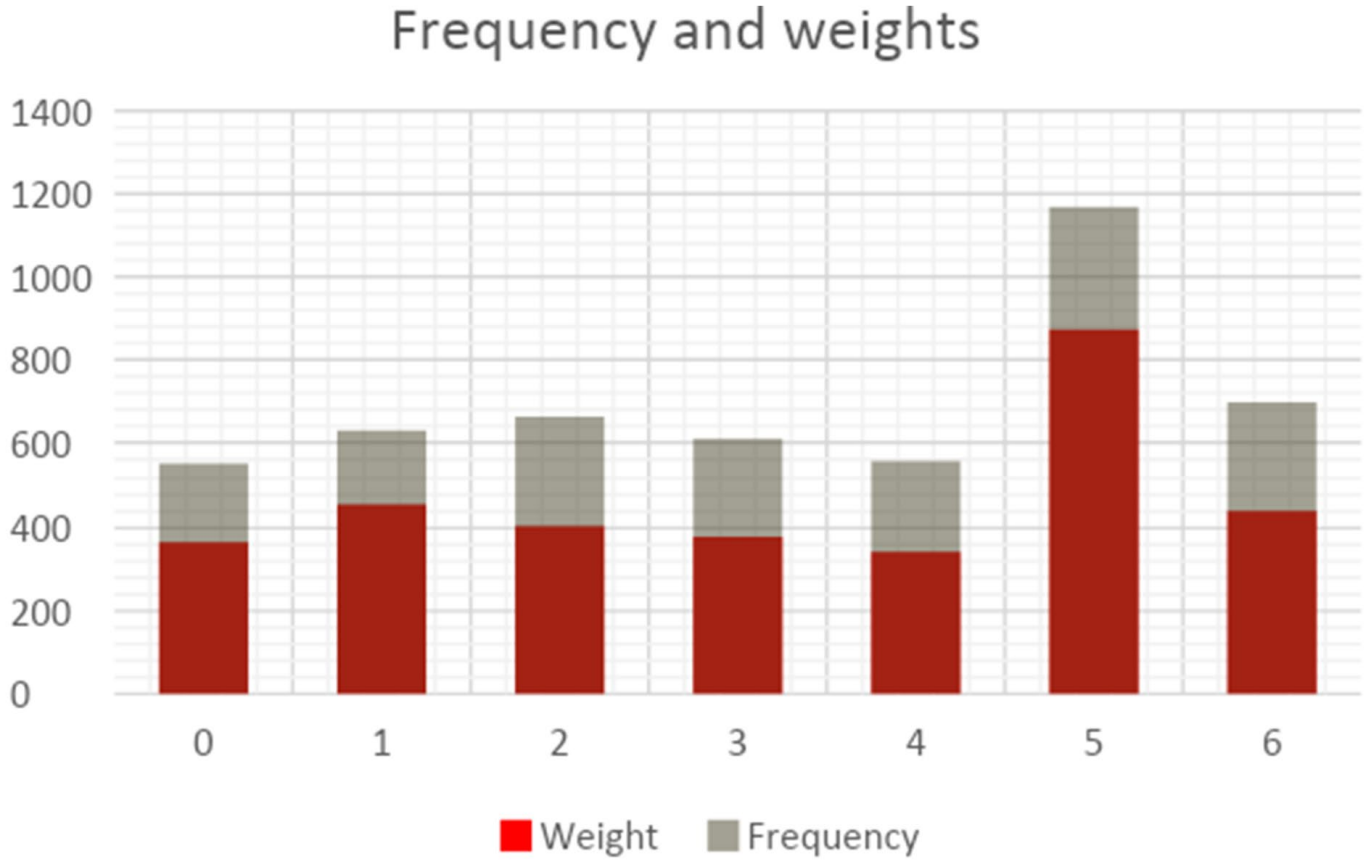
Frequency and weights (Scopus dataset).

Figure 4 displays the distribution of topics in the dataset. Two measurements, namely frequency and weights, are used. The term “frequency” pertains to the quantitative representation of the instances of certain topics such as topic 5, which has been documented occurring 1,169 times. The weight of a topic offers essential details on its uniqueness. Within the framework of topic modeling, each abstract is assigned a principal topic, while having the potential to include aspects from other topics. The chart shown demonstrates that the weight-to-frequency ratio stays rather stable across each topic, suggesting a degree of uniqueness among them.

Table 4 provides the mean values of citations and Altmetric mentions. Topic 0 consistently receives the most scientific and Altmetric impact in both scenarios. The topic has attracted significant attention and significance in light of the Covid-19 epidemic spanning from February 2020 to 2022. Furthermore, the research topics were regarded adequate for addressing the study methods and findings in articles published throughout this period.

**Table 4:** Citations and altmetric mentions.

	Topic 0	Topic 1	Topic 2	Topic 3	Topic 4	Topic 5	Topic 6
Citations	52.54	30.93	12.18	13.07	16.71	15.65	11.19
Altmetric	66.74	42.18	20.00	4.87	6.52	0.36	1.07

Table 5 highlights the 10 most often cited scholarly articles that relate directly to the field of crime, after the exclusion of works that were not within the scope of this study. It is worth noting that of the most frequently cited publications, only one received Altmetric mentions.

**Table 5:** Most cited papers.

Title	Citations	Topic	Altmetric mentions
Human and Machine Recognition of Faces: A Survey	1933	5	–
Youth risk behavior surveillance - United States, 2017	1,091	1	–
Accessorize to a crime: Real and stealthy attacks on state-of-the-art face recognition	569	5	–
Age synpaper and estimation via faces: A survey	544	5	–
Intelligent distributed surveillance systems: A review	516	5	–
Total confinement: Madness and reason in the maximum-security prison	351	3	–
SCface - Surveillance cameras face database	311	5	–
Police killings and their spillover effects on the mental health of black Americans: a population-based, quasi-experimental study	250	0	2,553
The work of watching one another: Lateral surveillance, risk, and governance	266	2	–
On the run: Wanted men in a Philadelphia Ghetto	267	3	–

Table 6 presents the publications that have been most often cited on Altmetric sources. While all of these papers have received citations, their cumulative citation count is rather modest. Regarding the selection of topics, it is evident that topic 5 exhibits the highest frequency among the most frequently cited papers, yet it is notably absent from the list of most often mentioned articles in Altmetric sources. In contrast, topic 2 has a much higher prevalence in the papers that get the highest number of mentions on Altmetric sources, while being less often cited in the most frequently cited publications.

**Table 6:** Most mentioned papers.

Title	Altmetric mentions	Topic	Citations
Police killings and their spillover effects on the mental health of black Americans: a population-based, quasi-experimental study	2,553	0	250
Leveraging Deep Learning and SNA approaches for Smart City Policing in the Developing World	508	2	8
Real-time suicide mortality data from police reports in Queensland, Australia, during the COVID-19 pandemic: an interrupted time-series analysis	259	0	41
Policing and public health—strategies for collaboration	84	2	22
Biometrics and public health surveillance in criminalized and key populations: policy, ethics, and human rights considerations	62	3	7
Mapping the rise of digital mental health technologies: Emerging issues for law and society	58	2	12
Consent and criminalisation concerns over phylogenetic analysis of surveillance data	37	2	7
Pandemic Surveillance and Racialized Subpopulations: Mitigating Vulnerabilities in COVID-19 Apps	33	2	6
New tools, old abuse: Technology-Enabled Coercive Control (TECC)	30	4	
The impact of license plate recognition technology (LPR) on trust in law enforcement: a survey-experiment	21	4	6

### 4.2 Public opinion (X/Twitter dataset)

The X/Twitter data was obtained through the Twitter API for academic research, granting comprehensive access to the platform’s database, spanning from its inception in 2006–2022. However, it’s crucial to acknowledge that the data and subsequent findings are constrained by the parameters of the search query employed and the preprocessing steps undertaken.

**Figure 5:**
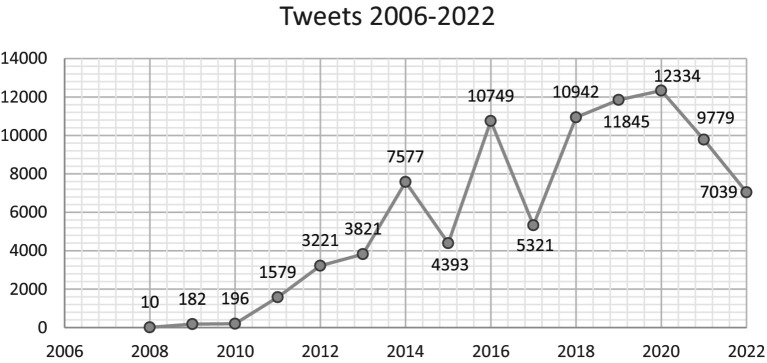
Tweets 2006–2022.

Figure 5 illustrates the distribution of tweets across the duration of the study. The numerical value had a significant rise throughout the time frame from 2008 to 2014, which aligns with the rise of X/Twitter as a popular social media platform. Hence, there is a noticeable change in the frequency of tweets related to this issue.

**Figure 6:**
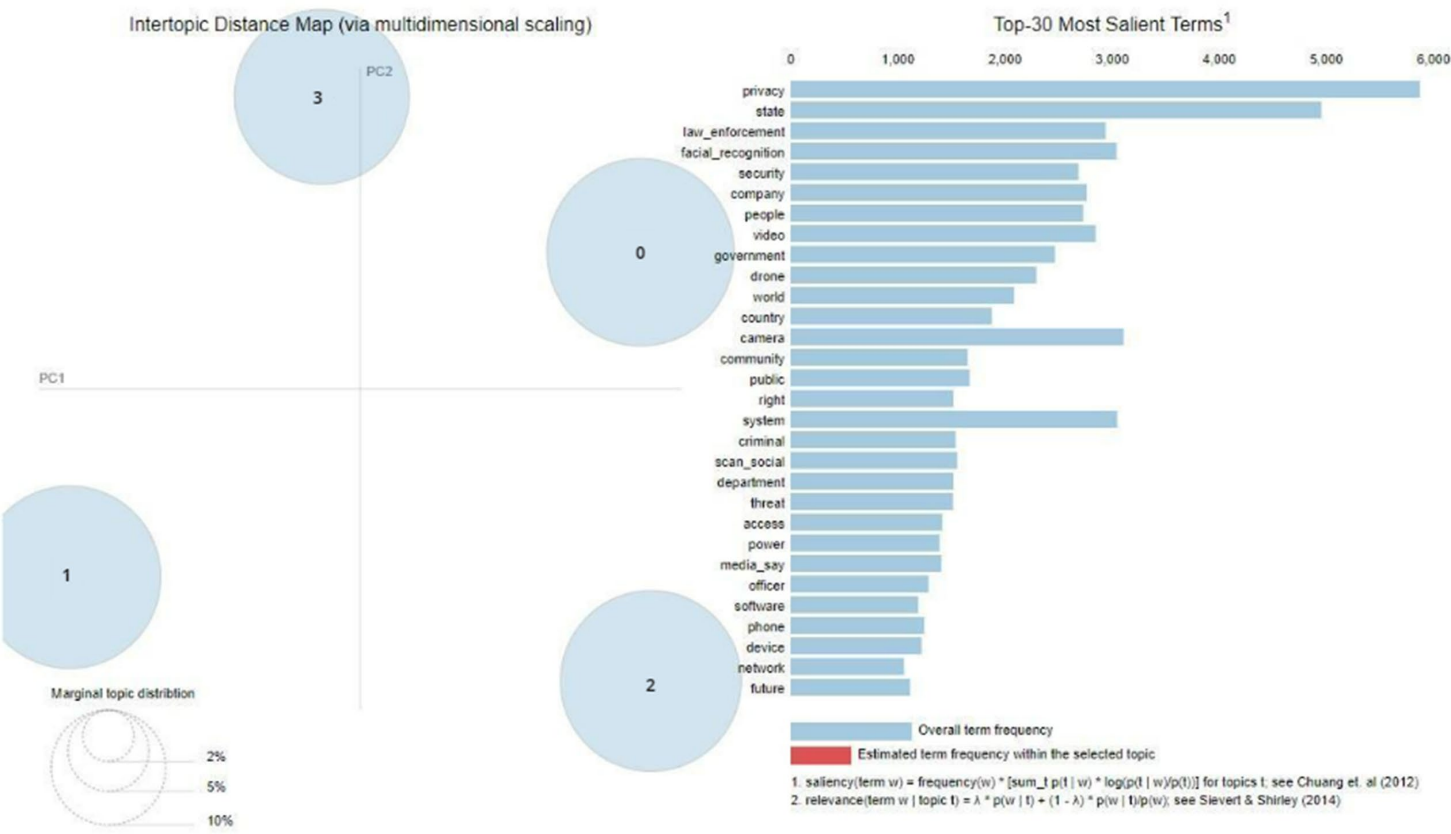
Topics from X/Twitter.

The outputs of the topic modeling technique applied to the X/Twitter dataset are shown in Figure 6. This analysis uncovers four unique topics that are linked to different aspects of surveillance technology. The whole information was analyzed to identify frequently recurring terms, including *privacy, law enforcement, face recognition, state,* and *security*. The figure also effectively demonstrates the division of these topics on the map, highlighting their clear classification without any instances of overlap. Table 7 displays the aforementioned topics in conjunction with their corresponding most prominent keywords.

**Table 7:** Topics (X/Twitter).

Number	Label	Keywords
Topic 0	Physical surveillance	Facial_recognition, video, company, people, drone, access, power, officer, future, report, protest, footage, today, policing, social_media
Topic 1	Policing & monitoring (Virtual and physical realm)	Law_enforcement, security, community, right, crime, software, network, force, system, amazon_pushes_facial_recognition, abuse, policing, secret, search, information
Topic 2	Governmental & military implementation	State, government, camera, public, criminal, agency, stingray, vast_power, crime, track, program, concern, biometric, digital, black
Topic 3	Privacy: Personal implications	Privacy, world, system, country, scan_social, department, threat, media_say, local, phone, device, citizen, family, misuse, camera

**Figure 7:**
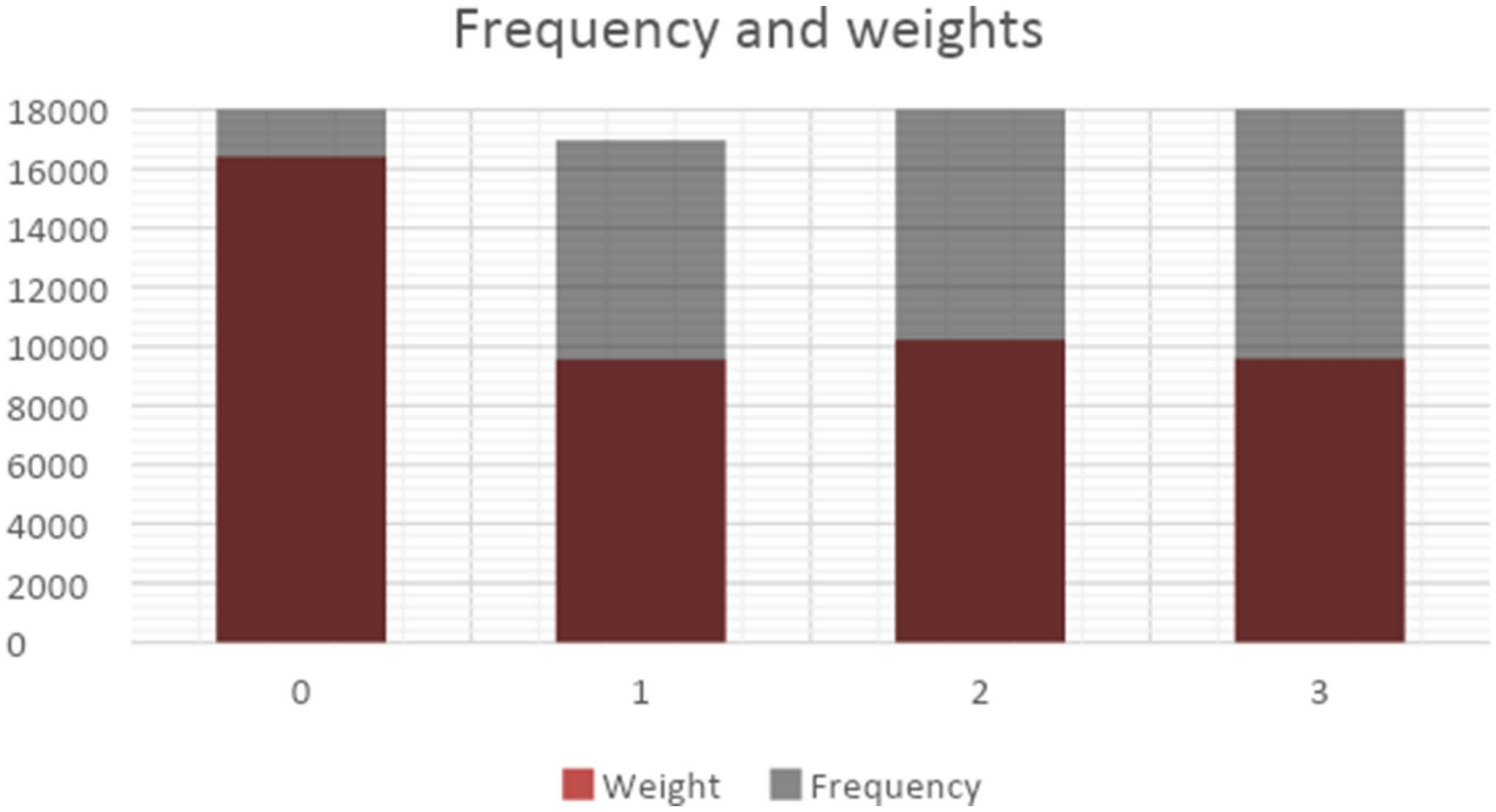
Frequency and weights (X/Twitter).

The X/Twitter dataset revealed four distinct topics. Topic 0 focuses on the use of monitoring in physical and public areas, particularly in relation to events like the Black Lives Matter movement, demonstrations, and the effective use of surveillance technologies in law enforcement. Topic 1 focuses on the execution of policing methods in both virtual (online) and physical realms. This includes the usage of software, the development of security solutions, and cooperation with organizations such as Amazon. In contrast, Topic 2 focuses on the use of surveillance technologies by governments and the military. This is highlighted by terms such as stingray, biometric, state, government, and military, with the military being included in the Topic 2 dataset. Finally, Topic 3 pertains to the issue of privacy issues. Figure 7 displays the topic frequency and weights, showing that Topic 0 has the greatest frequency, while Topics 1, 2, and 3 have similar sizes.

Table 8 displays the impact evaluation of several topics, showcasing indicators pertaining to the average count of likes, replies, and retweets. The data reveals an even distribution of likes across Topic 0, Topic 1, and Topic 2, however tweets linked to Topic 3 get comparatively less likes on average. Regarding the number of replies, Topic 2 has the greatest average count, whereas Topics 0, 1, and 3 have similar levels of engagement. When considering the number of retweets, it appears that Topics 1 and 2 exhibit the greatest average counts, while Topics 0 and 3 tend to acquire a lower number of retweets.

**Table 8:** Likes, replies and retweets.

	Average likes	Average replies	Average retweets
Topic 0	8.53	0.36	3.53
Topic 1	8.74	0.35	4.32
Topic 2	8.70	0.57	4.28
Topic 3	5.87	0.34	3.29

Table 9 provides a summary of the 10 tweets that have obtained the most likes in the dataset. Four of these tweets are classified as Topic 0, three as Topic 1, two as Topic 2, and one as Topic 3. Each topic is shown, but with different quantities. The tweets clearly demonstrate a significant emphasis on facial recognition, both its use by law enforcement agencies and the implications for the business sector, particularly corporations such as Amazon and IBM.

**Table 9:** Most liked tweets.

Tweet	Topic	Likes
Cool tweet. Will you commit to stop selling face recognition surveillance technology that supercharges police abuse? https://t.co/DfnAhyw2PW	0	109,717
BREAKING: California just blocked police from using face recognition surveillance on body cams. Let this be a warning to the companies and police departments rushing to adopt this dystopian technology: We will defend our right to privacy.	1	19,757
Shout out to @IBM for halting dev on technology shown to harm society. Facial recognition is a horrifying, inaccurate tool that fuels racial profiling + mass surveillance. It regularly falsely ID’s Black + Brown people as criminal. It should not be anywhere near law enforcement. https://t.co/SOU670yRVc	2	15,684
If you are considering Amazon’s Ring, please beware multiple privacy &amp, security concerns, including: Growing surveillance partnership w/police forces Third party trackers on its app Data breaches &amp, hackers infiltrating user homes via Ring camera https://t.co/d4BNIZmDtn	3	9,638
NEW: Major corporations are bankrolling police foundations across the U.S., helping them to buy surveillance tech, body armor and weaponry. https://t.co/8fUutXNB61 #BillionairesBackTheBlue https://t.co/u8G7zyLGuf	1	9,436
BREAKING: Detroit police just admitted what we have known all along — face recognition surveillance technology does not work. https://t.co/GDtfN3hTbb	1	8,449
“Another Bombshell: True The Vote’s Catherine Engelbrecht submitted multiple records requests to Yuma County for video surveillance and data from their drop boxes. She was told that this information no longer exists. If true, this is a crime. Some people need to be locked up.”	1	7,084
It is crucial to bear in mind that the industry’s problem is not data “protection,” it is data COLLECTION. Mass surveillance must be recognized as a crime, not a business model. https://t.co/L7Bu9otMWt	0	6,898
#AI based #FaceMaskViolationEnforcement is being rolled out by TS police. Leveraging ComputerVision &amp, #DeepLearningTechnique being implemented on surveillance CCTVs across the cities is #FirstOfItsKind in INDIA. Shall be enabled shortly across the 3Commissionerates *Hyd,Cyb&amp,Rck. https://t.co/hGwvq9cvsE	0	6,062
Breaking: NSA conveniently wipes out ‘01-‘07 mass surveillance data from warrantless &amp, unconstitutional STELLAR WIND program violating court orders in ongoing lawsuits against NSA. Obstructs justice. Destroyed criminal evidence against itself. No accident. https://t.co/jVWqVJ0F2Y	2	5,817

Regarding the tweets that received the highest number of retweets and replies, it was shown that they were closely associated with the tweets that received the most likes, as shown in Table 10. A few further instances are provided below.

**Table 10:** Key differences between two datasets.

	Key differences between Scopus and X/Twitter
Identified topics	7 topics (Scopus) vs. 4 topics (X/Twitter)
Privacy and Security	Higher ratio of altmetric mentions than citations
Applications for surveillance technology	Lowest number of altmetric mentions fourth most cited
E-health and disease management	Topic with highest impact on Scopus, not found in Twitter dataset
Military implementation	Low impact on Scopus, high impact on X/Twitter

*“After receiving complaints from the public, police near Minneapolis used high-tech surveillance drones last week to check if sunbathers at a lakeside beach were breaking the law by going nude or topless.* *https://t.co/S37FPYqwNk**”*

This tweet featuring the use of surveillance drones was categorized into topic 0 and got 423 replies, 397 likes, and 132 retweets.

*“Automating Racism: Police in China are using A.I. to mark/track ethnic minority Uighurs across the country. It’s the first known example of facial recognition being used intentionally by a government to racially profile and a massive ethical leap for A.I.* *https://t.co/QWvbPIj84z* *”*

This tweet emphasizes the use of extensive surveillance throughout the whole state by the autocratic government in China and demonstrates the consequences of such operations. The tweet received 2,931 retweets, 3,184 likes, and 174 replies, placing it under topic 2.

### 4.3 Connecting the dots

Topic modeling was used to evaluate tweets and academic publications. The Scopus and X/Twitter datasets identified seven and four topics, respectively. As can be seen from Table 10, the observed difference indicates a more complex and varied conversation around surveillance technologies in scholarly writing when compared to social media.

The research papers have discovered many significant topics, with Topic 0 (E-health & illness management) being especially remarkable. These topics highlight aspects that are not present in the X/Twitter dataset. Furthermore, both databases included diverse viewpoints on privacy, including those from the corporate sector and concerns at the social level.

Topic 3, which focuses on privacy and personal implications, received little attention on X/Twitter and ranked as the fifth most referenced and discussed topic on Scopus. This suggests that its influence was relatively restricted in both platforms. Topic 2, which focuses on privacy and security in the market and private sector, had the third highest number of mentions but the sixth highest number of citations among researchers. This indicates a divergence in attention between metrics related to citations and mentions. According to the Scopus dataset, Topic 6 (Defense & military implementation) was found to have the lowest number of citations, yet Topic 5 (Applications for surveillance technologies) had the fewest altmetric mentions. This highlights the disparities between academic citations and popular conversation.

It is noteworthy that Topic 2 (Governmental & military implementation) had the most significant influence on X/Twitter. On the other hand, Topic 1 (Policing & monitoring - Virtual and physical world) received the maximum number of retweets, but there was only little variance compared to Topic 2. In contrast, the topic that received the highest number of citations and mentions in Scopus was subject 0, which focused on E-health and illness management.

## 5 Discussion

The advent of digital tracking technologies, especially within the context of the COVID-19 pandemic, has resulted in an expanding field of research centered on issues pertaining to personal privacy. Nevertheless, the existing body of research has mostly concentrated on theoretical evaluations and opinion-based articles, leading to an absence of empirical evidence. This study provides a significant scholarly addition by presenting empirical findings that reveal people’ perspectives on surveillance within the context of the COVID-19 pandemic. Our research investigated the public’s views of surveillance technology on social media platforms and within research communities using topic modeling approach. We collected data from X/Twitter and Scopus for our analysis. Consequently, we have determined the prevailing topics found in these distinct corpora. In the following conversations, we examine the theoretical and practical contributions that have arisen from these insights. In addition, we analyze the consequences of these advances, tackle the limitations revealed throughout the study, and delineate new avenues for further research.

### 5.1 Theoretical and practical implications

The Panopticon metaphor, a recurring construct in the field of surveillance research, has been subject to critical examination from both theoretical and practical perspectives (McCahill, 2002; Galič et al., 2017; Ceccato, 2019). The analysis of distinct surveillance devices, ranging from traditional cameras to cutting-edge technology such as drones and satellites, is consistent with previous scholarly inquiries (Cayford and Pieters, 2020). Nevertheless, the changing environment goes beyond tangible objects and include the wider range of data collection conducted by authorities, particularly via social media and internet tracking. This statement highlights the rise of sousveillance, which refers to the use of commonplace technology to monitor and track their users (Ceccato, 2019). This aligns with the patterns identified in our empirical research. Furthermore, this is consistent with scholarly output concerning ethical AI which include pertinent issues such as privacy requirements, anonymisation and consent (Fontes et al., 2022).

Current conversations include several aspects of surveillance, including the unintentional influence of prevalent technologies like smartphones and social media in reshaping surveillance strategies. The term ‘Panopticommodity,’ introduced by Galič et al. (2017), aligns with our research, emphasizing the implicit collection of data and the passive acceptance of users. Such data is as debated by Fontes et al. (2022) crucial for high-quality results in AI-driven systems which can positively affect accuracy, efficiency and fairness in such systems. That said, data quality is just as important as quantity so such prerequisites should not be used as arguments for mass-surveillance. As stated by Fontes et al. (2022) several factors and the views of different stakeholders should be considered to ensure that the benefits outweigh the costs. An increase in data quantity enhances system preparedness for delivering high-quality outcomes, thereby positively affecting accuracy, efficiency, and fairness in user treatment. However, ensuring the reliability of outputs necessitates the utilization of not just larger volumes but also highly representative data, emphasizing the critical importance of data quality alongside quantity.

The issue of privacy, as emphasized in Topic 3 (Privacy: Regulation & Legislation) in relation to datasets, aligns with the broader public discourse around surveillance (Cayford et al., 2020). Once again connecting to the contemporary discussion on AI and ethics in a surveillance context as shown by Fontes et al. (2022).

The reciprocal characteristic of contemporary surveillance, which encompasses both sousveillance and surveillance, is consistent with the Deleuzian interpretation elaborated in previous scholarly works (Caluya, 2010). The phenomenon of users sharing data while being watched by advanced technical systems represents a significant and transformative change. The empirical data demonstrate the widespread use of various surveillance technologies, including standard cameras, drones, and face recognition software. This highlights how complex surveillance environments blur traditional boundaries and expand surveillance beyond limited areas (McCahill, 2002). As viewed by Saheb (2023), AI applications in particular can have unforeseen or even malignant consequences making their implementation more hazardous, especially due to concerns for the privacy and integrity of citizens. Meanwhile, the public acceptance of surveillance technology relies on trust, privacy and technological understanding (Fontes et al., 2022). Naturally, such issues are pertinent in the case of AI which is a complex in its nature and thus can be difficult to grasp.

Theoretical perspectives include actor-network theory, which has significant importance in the field of information systems, highlighting the widespread incorporation of technology in everyday existence. In the midst of technological progress, tracking has become widespread worldwide, as shown by the growing attention in both academic research and public discourses (Brayne, 2017). The authors Galič et al. (2017) have noted that power dynamics undergo changes that reflect shifts in socio-technological environments. The significance of the Panopticon metaphor endures despite the substantial advancements in technology since its creation. In our modern, technologically interconnected society, when Google, Facebook, and governmental institutions assume surveillance functions, the metaphor continues to symbolize the concept of the unseen gaze, a metaphor even more relevant as AI is integrated into the infrastructure of modern societies (Heilinger, 2022). The results of our study support the ongoing significance of the Panopticon in shaping our understanding of modern surveillance technologies, shedding light on the expanding technical range and social consequences.

This study primarily focuses on the improved processes and technologies used for thorough data collecting and analysis. The significance of refining data retrieval and analysis procedures across many study disciplines becomes apparent at a time characterized by the rapid growth of online data repositories and increased human involvement in digital realms. Significantly, in the field of informatics, which investigates the complex relationships between people and technology, the enhancement of these approaches becomes particularly crucial. Furthermore, the study presents empirical findings that reveal a significant difference between researchers and the general public on the key aspects of surveillance technology. This mismatch provides a significant distinction, which may be valuable for policymakers and lawmakers in defining priorities that represent the specific interests of different stakeholders. The comprehension of prominent themes in debates and dissemination has significant importance due to the pervasive impact of these stakeholders in political discourses. This understanding plays a crucial role in guiding policy discussions and decision-making processes.

### 5.2 Limitations and future research

The process of topic modeling requires the use of human judgment and assessment, which aligns with the interpretivist research paradigm. The level of fairness in computer-based methodologies prompts reflection, considering their dependence on programming languages, data sources such as X/Twitter, Altmetric, and Scopus, as well as the biases, experiences, and expertise of researchers. The lack of autonomy in the scripts used gives rise to inherent limitations, particularly in the selection of language and sources, which may introduce bias into the data and subsequent analysis. Although the positivist tradition employs quantitative methods such as web scraping, topic modeling, and established statistical measurements to reduce biases, it is important to interpret quantitative metrics like citation counts or Altmetric mentions in a contextualized manner, considering their strengths and weaknesses. Altmetric metrics, however useful for assessing research interest outside the academic realm, have limitations in terms of disregarding mentions that lack appropriate citations or include inaccurate DOIs or URLs (Cronin and Sugimoto, 2014; Hammarfelt, 2014; Holmberg et al., 2020).

In brief, the computer-based methodologies used in this study do not provide a comprehensive analysis of the mechanisms or rationales for technology utilization, which are often explored using qualitative approaches (Kaplan and Maxwell, 2005). However, the tweets and academic articles that have been retrieved provide insights into these dimensions. Specifically, the tweets that had the highest number of interactions emphasized concerns around surveillance at the state level and intrusion by the government. Nevertheless, computer-based approaches provide exceptional benefits in terms of accessing and evaluating huge amounts of data in a consistent manner, in contrast to human observers who are prone to discouragement and subjectivity.

The key methodology used in this work utilizes machine learning techniques to cluster words and identify themes within text corpora (Cain, 2016). Although this methodology provides useful insights, it is limited in its ability to assess sentiment related to specific topics hence restricting an in-depth understanding of the emotional nuances linked with each theme. Subsequent research efforts could improve this study by including sentiment analysis, enabling a thorough investigation of the prevailing emotions among the selected topics. By establishing a correlation between sentiment and topics, researchers may uncover the emotional impact of various subjects, thereby enhancing the understanding obtained from the data. The authors Müller et al. (2016) emphasized the importance of integrating sentiment analysis as a means to address this constraint in topic modeling approaches.

Furthermore, embracing a longitudinal approach may reveal the temporal dynamics of these topics providing insight into their progression over a period of time. By using statistics from X/Twitter and Scopus, researchers can investigate the impact of social events on the frequency of conversation around different subjects. An analysis of the variations in topic trends pertaining to significant events, such as scandals or instances of whistleblowing, has the potential to provide insight into the influence of these events on the discourse within research communities and social media platforms (Kastrati et al., 2015).

In addition, future inquiries may broaden the range of analysis by examining data derived from different sources. Conducting comparative research on various social media sites, such as Facebook, Reddit, and Quora, might clarify the unique characteristics of each platform in the discourse and spread of topics linked to surveillance technologies. An area of potential interest for bibliometric and scientometric studies might be the examination of variances in research paper subjects across distinct academic domains. The use of comparative analysis offers potential in identifying both commonalities and differences in the discourses surrounding topics across diverse platforms and academic fields.

## Data availability statement

The original contributions presented in the study are included in the article/Supplementary material, further inquiries can be directed to the corresponding author.

## Author contributions

KK: Conceptualization, Data curation, Formal analysis, Methodology, Software, Visualization, Writing – original draft, Writing – review & editing. FD: Conceptualization, Methodology, Project administration, Supervision, Writing – original draft, Writing – review & editing.
