# Supplementary material for: Exploring the surveillance technology discourse: a bibliometric analysis and topic modeling approach
Source: Front Artif Intell. 2024 Jun 6;7:1406361. doi: 10.3389/frai.2024.1406361 (PMC11188983; doi:10.3389/frai.2024.1406361)
Supplement: Supplementary file 1 [file Data_Sheet_1.docx]

**Supplemental file: Topic modeling code snippets.**

**Scopus:**

vectorizer = CountVectorizer(

analyzer='word',

min_df=15, # minimum reqd occurences of a word. Adjust accordingly!

stop_words='english', # remove stop words

lowercase=True, # convert all words to lowercase

token_pattern=

'[a-zA-Z0-9]{3,}', # Which type of tokens get accepted and minimum length (3).

# max_features=50000, # max number of uniq words. Didn't use this one!

)

number_topics = 7

lda_model = LatentDirichletAllocation(

n_components=number_topics, # Number of topics

max_iter=70, # Max learning iterations

learning_method='online',

learning_decay=0.9,

random_state=42, # Random state

batch_size=512, # n docs in each learning iter

evaluate_every=-1, # compute perplexity every n iters, default: Don't

n_jobs=-1, # Use all available CPUs

)

**Twitter:**

vectorizer = CountVectorizer(

analyzer='word',

min_df=100, # minimum reqd occurences of a word. Adjust accordingly!

stop_words=stop_words, # remove stop words

lowercase=True, # convert all words to lowercase

token_pattern=

'[a-zA-Z0-9_#]{5,}', # Which type of tokens get accepted and minimum length (3).

# max_features=50000, # max number of uniq words. Didn't use this one!

)

data_vectorized = vectorizer.fit_transform(data_lemmatized)

#Set number of topics here. For deciding learning decay and max_iter I use Gridsearch.

number_topics = 4

# Build LDA Model

# Remember! These are the parameters I ended up with in my study. Needs to be adjusted!

lda_model = LatentDirichletAllocation(

n_components=number_topics, # Number of topics

max_iter=80, # Max learning iterations

learning_method='online',

learning_decay=0.9,

random_state=42, # Random state

batch_size=256, # n docs in each learning iter

evaluate_every=-1, # compute perplexity every n iters, default: Don't

n_jobs=-1, # Use all available CPUs

)
